# Application of Gas Chromatographic Retention Indices to GC and GC–MS Identification with Variable Limits for Deviations Between Their Experimental and Reference Values

**DOI:** 10.3390/molecules30244706

**Published:** 2025-12-09

**Authors:** Igor G. Zenkevich

**Affiliations:** Institute for Chemistry, St. Petersburg State University, 198504 St. Petersburg, Russia; izenkevich@yandex.ru; Tel.: +7-(812)-428-4045

**Keywords:** gas chromatography, retention indices, reference values, correction of experimental data, identification, databases

## Abstract

The potential of a new algorithm for comparing experimental and reference values of gas chromatographic retention indices (RIs) is discussed. This algorithm is designed to minimize significant elements of uncertainty typical of numerous contemporary recommendations, primarily, the fixed limiting values of permissible deviations between experimental and reference RI-values, ΔRI = (RI_ref_ − RI_exp_). The algorithm proposed implies the calculation of deviations, ΔRI, for the most reliably identified constituents of multicomponent mixtures in different parts of chromatograms with known reference RI values, followed by calculation of coefficients of regression equations ΔRI = (RI_ref_ − RI_exp_) = *a*RI_exp_ + *b* for both of the reduced sets of analytes. This equation allows for the recalculation of experimentally determined RIs into corrected values RI_corr_ = RI_exp_ + ΔRI, which means replacing the fixed “global” limits with data-dependent adaptive thresholds for different constituents of multicomponent samples. Such an algorithm makes it possible to use reference RI values for semi-standard nonpolar polydimethylsiloxane phases (with 5% phenyl groups and others) for the comparison with data determined with standard nonpolar polydimethylsiloxanes and vice versa, as well as to minimize the influence of possible erroneous reference RI data. It is applicable both to statistically processed reference data and to results of single measurements. Both of these kinds of reference data are known and presented in contemporary RI databases, e.g., in the NIST RI database.

## 1. Introduction

At present, gas chromatography–mass spectrometry (GC-MS) appears to be the most effective and widely applicable hyphenated technique for the analysis of organic compounds in complex mixtures. This is due to the availability not only of modern equipment, but also of detailed and well-systemized informational support. It includes databases of standard mass spectra (electron ionization, 70 eV) and gas chromatographic retention indices (RIs [1]) on standard nonpolar (polydimethylsiloxanes) and polar (polyethylene glycols) stationary phases. An example of a combination of these parameters is the NIST mass spectral database [2], which has been supplemented with RIs since 2005. The last version of this database (2023) contains mass spectra of 347,000 compounds and the RIs of 153,000 compounds.

The database’s application efficiency is determined not only by the number of objects included, but by the algorithms used for comparing experimental and reference (library) data, both mass spectra and RIs. Many of the previously proposed mass spectrometric algorithms are currently only of historical interest because the most widely used is the algorithm proposed by employees of the Finnigan Co. in 1978 [3] and its subsequent modifications. Its essence is as follows: each mass spectrum under comparison can be represented as a vector in an *N*-dimensional space, where *N* is the number of signals (in other words, the maximal *m*/*z* value). The numerical expression of the mutual correspondence of these vectors (other terms are similarity and match factor (MF), etc.) is the square of cosine of the angle (Θ) between them, 0 ≤ cos^2^Θ ≤ 1. For greater clarity, the normalization condition often transforms into 0 ≤ MF ≤ 100, or 0 ≤ MF ≤ 1000 (the maximal MF value corresponds to the maximal similarity of mass spectra, and the minimal MF value, to their complete dissimilarity). The normalization 0 ≤ MF ≤ 100 is sometimes called a percentage match, which is incorrect.

In contrast to multidimensional mass spectra, GC retention indices are one-dimensional analytical parameters (one number). It would seem that assessing the degree of coincidence of one-dimensional values should be much simpler compared to multidimensional values. However, this is not the case, and this problem remains unsolved to date. The experimental RI values cannot exactly match with reference data, because the former values are influenced by experimental errors, and the latter values, by interlaboratory irreproducibility. The reason for restricted interlaboratory reproducibility of RIs is their dependence on the conditions of GC analyses, primarily on temperature, even at the stationary phase. In turn, the temperature depends on the geometry of the chromatographic column and the amount of the stationary phase in it. In addition, the RI values depend on the ratio of peak areas of the analytes and reference compounds [4]. Hence, if we postulate any RI values as the reference information, RI_ref_, and compare the experimental RI_exp_ values with them, the differences, ΔRI, between them turn out to be inconstant. Theoretically, we can imagine the existence of limiting values for these differences, ΔRI_lim_. Combining all these premises, we can formulate the simplest condition of GC identification using retention indices:

ΔRI = |RI_exp_ − RI_ref_| ≤ ΔRI_lim_ (1)

If ΔRI > ΔRI_lim_, the identification must be considered impossible even at the “ideal” mutual correspondence of the mass spectra.

The values of ΔRI_lim_ depend not only on the nature of the stationary phase and specific conditions of the analysis, but also on the chemical nature of analytes and the features of the database used. In accordance with the long-established practice, the correctness of GC identification is most often illustrated by direct comparison (for visual perception) of the experimental and reference values; see, e.g., publications [5,6,7,8,9,10,11,12,13,14], but the number of examples is numerous.

Attempts have been made to create combined criteria for joint mass spectrometric and gas chromatographic identification. For example, Smith with coauthors [15] proposed the criterion *F* = *U*·MF as follows:

*U* = 1, if ΔRI ≤ 5,
1 − 0.05|ΔRI|, if 5 < |ΔRI| ≤ 20 = ΔRI_lim_
0, if |ΔRI| > 20,(2)

where MF is the mass-spectrometric match factor and *U* is the measure of correspondence of GC RIs. In other words, small differences, ΔRI, (≤5 index units) do not affect the results of mass-spectrometric identification, while large differences (>20 i.u.) lead to the elimination of this option from further consideration despite the best correspondence of mass spectra. Such conditions seem to be useful as very preliminary estimates, but it have obvious disadvantages, namely:

1. The choice of fixed value ΔRI_lim_ = 20 i.u. seems to be illogical because the differences between experimental and reference data can take both larger and smaller values depending on the chemical nature of analytes and types of stationary phases under comparison;
2. Condition (2) implies the symmetrical distribution of ΔRI, in other words, the equally probable deviations of experimental and reference data both at ΔRI < 0 and ΔRI > 0, but in the general case, it is not obvious.

**Figure 1:**
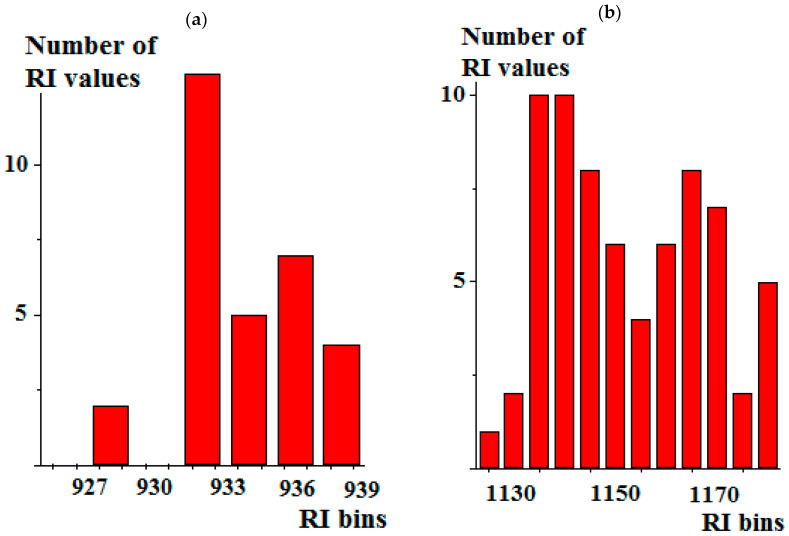
Histograms illustrating the distribution of reference RI values for (**a**) 2,6-dimethyloctane and (**b**) 1,2,3,4-tetrahydronaphthalene (tetralin) on standard nonpolar polydimethylsiloxane stationary phases [2]. The bin sizes are (**a**) 2 i.u. (index units) and (**b**) 5 i.u. The average RI values are (**a**) 933 ± 3 and (**b**) 1152 ± 15.

**Figure 2:**
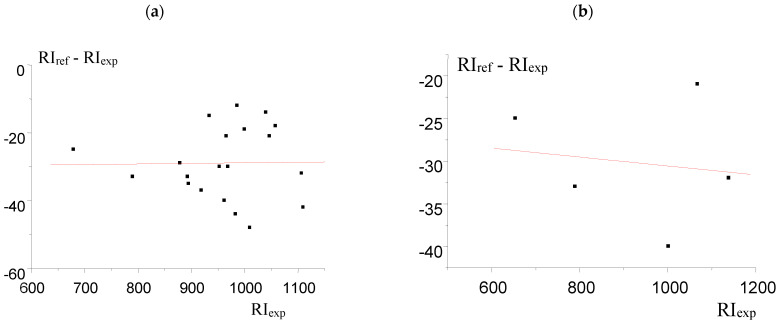
(**a**) Linear approximation of the differences ΔRI_ref-exp_ = (RI_ref_ − RI_exp_) vs. RI_exp_ plots for the complete data set from [20]; reference data are taken from NIST database [2]. The parameters of the regression equation are indicated in the footnotes to Table 1; (**b**) the same data for the reduced data set for five alkylarenes selected from their total list [20].

**Figure 3:**
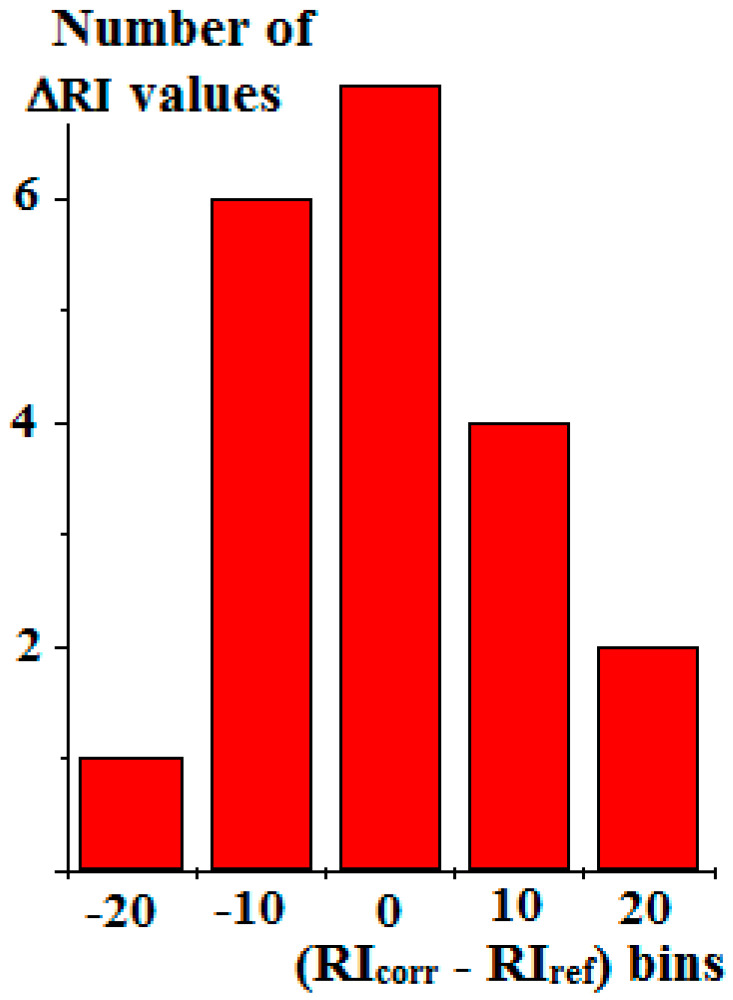
Histogram of the distribution of retention indices of alkylarenes [20] recalculated into RI_corr_ values. The bin size is 10 i.u. The average RI value of the data considered with their signs is 0 ± 11.

The differences in the real interlaboratory RI distribution can be best illustrated by histograms for 2,6-dimethyloctane (Figure 1a) and 1,2,3,4-tetrahydronaphthalene (tetralin), (Figure 1b). Both histograms are unsymmetrical, that is typical for GC retention indices and leads to the hypothetical incorrectness of conventional statistical data processing. Moreover, the histogram for tetralin allows us to identify the two-modality RI distribution, which is quite common, but the explanations for this can be difficult [16]. However, because other algorithms of data processing are too complicated, we have to calculate arithmetic averages together with their standard deviations, which gives 933 ± 3 for 2,6-dimethyloctane (a) and 1152 ± 15 for tetralin (b). The first compound illustrates the good interlaboratory RI reproducibility, but the second case is more typical of numerous organic compounds.

Such spread of RI values depends on the chemical nature of the analytes and determines the difficulties and uncertainties of their application [17,18]. It explains the use of arithmetic averages in combination with the corresponding evaluations of permissible deviations, commonly accepted standard deviations, <RI> ± *s*_RI_. According to the relationships of statistical processing, the intervals <RI> ± *s*_RI_ include about 68% of values of the initial data set; the intervals <RI> ± 2*s*_RI_, about 95%; and the intervals <RI> ± 3*s*_RI_, about 99.7%. In addition, the reference data presented without deviations are known, e.g., reference data for constituents of essential oils [19]. In the NIST database [2] and in some secondary data summaries based on [2], the RI spread historically is customarily characterized by MAD values (medians of absolute deviations). This means that the intervals <X> ± MAD contain only 50% of values of the initial data set. Furthermore, the number of single RI measurements (measured in one laboratory) in the last versions of the NIST database increased noticeably; such data have no measures of possible deviations at all.

The mentioned features of interlaboratory distributions of RI values explain the difficulties of selecting the criteria for comparing their experimental and reference values. Nevertheless, there is an actual need for creating the corresponding algorithm. The algorithm must have the following properties:

- It should not include any fixed limiting values of possible deviations between experimental and reference RI values;
- It should be applicable to asymmetrically distributed reference RI values;
- It should be applicable to both statistically processed reference data and the results of single measurements;
- It should be applicable to compare the RI values determined using columns with semi-standard stationary phases (terminology used in database [2]) with reference data for standard nonpolar stationary phases and vice versa;
- It should be applicable to RI values measured for both capillary and packed chromatographic columns at various temperatures without any artificial restrictions.

The novelty of the approach proposed implies, not the use of fixed global limits but adapted RI thresholds for deviations RI_exp_ − RI_ref_ during RI library searches. In addition, we can use such term like “database harmonization” (proposed by the reviewer), because this approach allows for partial compensation of the possible presence of non-optimal reference RI values (i.e., determined in non-optimal conditions) without requiring the reconstruction of whole databases. This paper discusses the attempt of creating an algorithm that meets all the above-listed conditions.

## 2. Results and Discussion

It is appropriate to start the discussion of an algorithm that meets the a priori requirements listed above not with theoretical consideration, but with a specific example. At the same time, this example illustrates the symbolism used. Here, and later in the tables, the symbols RI_exp_ are the values from original publications or experimental RI values, the symbols RI_ref_ are reference RI values from a database (with reference), Δ_ref-exp_ = RI_ref_ − RI_exp_ are the differences between reference and experimental values, and RI_corr_ are corrected experimental values calculated using the total data set, while RI_corr_ * are the values calculated using the reduced data set. Finally, Δ_corr-ref_ are the differences between corrected and reference RI values. Every table from nos. 1–4 is finalized with the indication of average standard deviations of reference RI values, *s*_RI_, average RI difference, Δ_ref-exp_, and average differences, Δ_corr-ref_.

### 2.1. Optimization of Comparing the Experimental and Reference Values of GC Retention Indices

To start the discussion, let us select the example of GC retention indices of 20 compounds of the same chemical class (alkylarenes), listed in Table 1, but measured under very specific conditions, namely, packed column with semi-standard hydrocarbon phase Apiezon L (15% on Celite C-22) at 100 °C [20]. According to contemporary concepts, such conditions are completely outdated because too high a content of the stationary phase leads to too high a temperature for chromatographic separation.

As a result of using such obsolete analytical conditions and semi-standard phases, all the experimental RIs exceed contemporary reference RI values [2] by approximately 30 i.u. (differences vary from 12 to 48 i.u.). The values of Δ_ref-exp_ = RI_ref_ − RI_exp_ for all the compounds are presented in Table 1. The comparison of these values requires taking into account not only their random variations, but also systematic differences caused by the nonequivalence of the determination conditions. This means that we must take into account the possible dependence of amendments Δ_ref-exp_ = RI_ref_ − RI_exp_ on the values of RI_exp_ themselves:

Δ_ref-exp_ = (RI_ref_ − RI_exp_) = *a*RI_exp_ + *b* (3)

The plot of the dependence (3) (swarm of dots) is shown in Figure 2a. It should be especially noted that the small absolute value of correlation coefficient (*R*), is −0.02 in this case. This is not a sign of the unsuitability of such statistical processing, but the objective feature of the data array. The low *R*-value means that any mutual variations in experimental and reference RIs are expressed to a low extent, but both sets of data are strongly influenced by random RI errors. In such cases, the sum of residuals (*S*_0_) appears to be a more informative criterion than correlation coefficients.

Thus, we come to the following conclusions. Firstly, the Δ_ref-exp_ values show significant scatter. Hence, secondly, there are no reasons to approximate these data by polynomials of higher orders. Such a conclusion is explained by the small number of points (RI values) and their wide dispersion; it may be acceptable for linear regression, but insufficient for higher-order polynomials.

Thirdly, the standard deviation of coefficient “*a*” of dependence (3) exceeds the absolute value the value of the coefficient itself, which means that there is a weak dependence of amendments Δ_ref-exp_ on RI_exp_ values. Finally, the determination of the parameters of Equation (3) (even at the small value of coefficient “*a*”) allows us to convert the experimental data published in [20] to the corrected RI_corr_ values used for comparisons with information from the contemporary database [2]:

RI_corr_ = RI_exp_ + Δ_ref-exp_ (4)

The values of the Δ_ref-exp_ correction are not constant (see Equation (3)) but depend on the conditions for experiments determining the retention indices and the features of the RI database.

All the RI_corr_ values are also listed in Table 1. As we can see, the average absolute values of differences Δ_corr-ref_ (9 ± 6) appeared to be three times smaller than the average absolute value of differences Δ_ref-exp_ (29 ± 10). It should be noted that the average corrected values ± standard deviations considered by taking the signs of the initial data into account differ from zero statistically insignificantly (0 ± 11). Their distribution can be additionally illustrated by the corresponding histogram (Figure 3). It is more symmetric than the initial set of ΔRI values, but small asymmetry of these parameters is typical for many organic compounds. Their normal distribution seems practically unattainable:

However, the correction of the experimental RI values for providing the possibility of their optimal comparison with the available reference data is only “half” of the problem. The second part is the evaluation of the permissible deviations of RI_corr_ values from RI_ref_ for accepting or rejecting the gas chromatographic identification. In the least-squares method, the formula exists for a so-called “corridor of errors” for evaluating the possible deviations of points from the regression equation *y* = *ax* + *b* [21]:

(5) $$S(x)\;=\;s_{y}\sqrt{\frac{\left(1-R^{2}\right)}{N-2}\times \left[1+\frac{{\left(a\;-\;<x>\right)}^{2}}{sx^{2}}\right]}$$

where *s_x_*^2^ = (<*x*^2^> − <*x*>^2^/*N*)/*N*, *s_y_*^2^ = (<*y*^2^> − <*y*>^2^/*N*)/*N*, and *R* = (<*xy*> − <*x*><*y*>/*N*)/(*Ns_x_s_y_*).

However, this relationship seems too complicated for routine calculations; simpler evaluations are preferable. If the dependence, ΔRI_corr_ = *f*(RI_exp_), is expressed rather weakly, (like it is in the example considered), we can accept ΔRI_corr_ const. The first recommendation implies using the evaluations of the standard deviations of ΔRI_corr_, namely, *s*_ΔRIcorr_. Most ΔRI_corr_ values do not exceed ΔRI_corr_ + *s*_ΔRIcorr_, while the values exceeding ΔRI_corr_ + 2*s*_ΔRIcorr_ can be excluded from consideration. The second way to evaluate the possible deviations is based on such a parameter as the “sum of residuals”, *S*_0_. For numerous examples (including those considered above), the following inequality is correct:

ΔRI_corr_ + *s*_ΔRI_ < *S*_0_ < ΔRI_corr_ + 2*s*_ΔRI_ (6)

This means that the RI_corr_ data for GC identification should be chosen within the intervals of RI_ref_ ± 2*S*_0_:

RI_ref_ − 2*S*_0_ < RI_corr_ < RI_ref_ + 2*S*_0_ (7)

The Δ_corr-ref_ values in Table 1 indicate that only one of the twenty compounds (1,2,3-trimethylbenzene) do not meet this criterion. Using the analogy with data statistical processing, we emphasize that if we cannot identify only one from the twenty analytes, it is similar to a confidence probability of identification of approx. 95%. Hence, it is a statistically acceptable result (95% of correct answers).

However, the illustration of data processing mentioned above looks like an artificial example because it implies the recalculation of the RI values of all analytes without exception (in the hypothetical condition that we know all of them a priori). In real analyses of complex mixtures, we do not know all of the analytes before analysis (to identify them is the final aim); usually, we can only identify several constituents using their specific mass spectrometric signs or preliminary chemical information. Hence, the application of the algorithm considered to real multicomponent mixtures requires modification.

### 2.2. Application of the Algorithm of Comparing the Experimental and Reference GC Retention Indices to Multicomponent Mixtures

This is a typical analytical task. Most often, complex multicomponent mixtures under analysis contain some simple constituents that can be unambiguously identified without special processing of GC data. These components are, for example, impurities in commonly used solvents and reagents, plasticizers (e.g., phthalates), and so on. Hence, we can just select these compounds for a comparison of their RI_exp_ and RI_ref_ values. The number of such “reference points” may not be so large, but it is important that they should be evenly distributed in different parts of the chromatograms (at the beginning, in the middle, and in the final part). Of course, if the samples under analysis contain a small number of constituents (e.g., 1–2), the preliminary analysis of artificial mixtures of similar compositions becomes necessary.

Continuing the consideration of the above-mentioned example (Table 1, Figure 2 and Figure 3), we can select from 20 alkylarenes only 5, namely, benzene, toluene (the first segment of the chromatogram), 1,3,5-trimethylbenzene (middle position), butylbenzene, and 1,2,4,5-tetramethylbenzene (the last part). All the selected compounds are marked in Table 1 in bold. After that, we should repeat the same mathematical operations as were performed for the complete data set (Equations (3), (4), (6) and (7)) for this reduced data set. Obviously, we obtain different values of the coefficients of the linear regression Equation (3) than for the full data set (see footnotes to Table 1).

The plot of the dependence (3) for the reduced data set is shown in Figure 2b. It illustrates slight variations in the angular coefficient “*a*” in Equation (3); the dependence becomes slightly ascending instead of slightly descending. The values of ΔRI_corr_ * = (RI_corr_ − RI_ref_) together with Δ_corr-ref_ * are marked with asterisks in Table 1 for comparison with the initial values of ΔRI_corr_ = (RI_corr_ − RI_ref_) and Δ_corr-ref_. However, the average value of Δ_corr-ref_ * (8 ± 6) appeared to be very close to Δ_corr-ref_ (9 ± 6). The same is true for the sum of residuals (*S*_0_): 10.8 and 8.4, respectively. This means that RI_corr_ * does not correspond to the RI_ref_ ± 2*S*_0_ intervals for only 1 compound of 20 (namely, for *tert*-butylbenzene), which is statistically acceptable (95% of correct results; see comments above).

An important feature of data processing should be noted here. The algorithm proposed implies the formation of the reduced RI data set from the initial array (this is the important element of its novelty). This reduced array must preserve the properties of the original data, primarily the spread of RI_ref_ − RI_exp_ values. The best criterion for this “reproducibility of properties” is comparing the values of correlation coefficients, *R*. In other words, if the *R*-value for the initial data set is small (which is most commonly the case), then the *R*-value for the reduced data set should be close to it. Figure 2 illustrates this feature: the *R*-value for the data plotted in case (a) is −0.02, while in case (b) it is −0.14. Moreover, if the R-value for the initial set of data is small, but increases significantly after reducing, this means that the reduced data set was chosen incorrectly.

It seems important to note that *R*-values illustrate the operations with RI data sets but cannot be used to characterize identification itself. It is known that *R*-values express the conformity of the dependence considered to a linear (or another) regression. However, this is not a problem that needs to be solved in our case. The final predestination of the algorithm considered is to evaluate the permissible deviations of RI_ref_ − RI_exp_ values from the regression line. Hence, the preferable parameter in the least-squares method is the function of such deviations, namely the sum of residuals, *S*_0_. Just the *S*_0_ values are recommended as the main criteria for applying the RI data for GC identification (Equation (7)). It should also be confirmed with Figure 2; the *S*_0_ value for case (a) is 10.8 i.u. (index. units), while for case (b) it is close to it (8.4).

The above reasoning can be illustrated by considering the RI values for 32 essential oil constituents, published by Engewald and co-authors [22] for a column with a standard nonpolar polydimethylsiloxane stationary phase, DB-1. The constituents of essential oils belong to different classes of organic compounds and, hence, such examples illustrate the capabilities of the algorithm well. The results of this data processing in comparison with the reference RI values from database [2] for identical phases are presented in Table 2. The reduced data set includes six compounds easily identifiable by mass spectra, namely, α-pinene, limonene, linalool, camphor, neral, and geranial (marked in bold). Similarly to Table 1, the parameters of all of the equations (RI_ref_ − RI_exp_) = *a*RI_exp_ + *b* for both the complete and reduced data sets are indicated in the footnotes to Table 2. The average value of the difference RI_ref_ − RI_exp_ for the complete data set is 13 i.u., and for the corrected reduced data set it is twice as low, 6 i.u.

The single compound with RI_corr_ values that do not meet condition (7) for both the complete and reduced data sets is isosafrole, 5-(1-propenyl)-1,3-benzodioxole. This is most probably due to the unreliable reference RI value for this compound in database [2]. Indeed, the RI value for isosafrole in [22] is 1357, while the reference value is 1327 ± 31 [2]. Such a large standard deviation is explained by uniting the RI values for so-called α- and β-isosafroles (*cis* and *trans* isomers) together. The RI value for the most often determined β-isosafrole (*trans-*isomer) is 1358 ± 6 (value from the author’s RI collection). Keeping this fact in mind, we obtain for this compound Δ_corr-ref_ * = −1 instead of +30. After this correction, the RI value for isosafrole (the last eluted compound) can be included in the reduced data set. It is an additional illustration of the efficiency of the suggested approach.

The same example of 32 essential oil components can be considered by using another database of reference information for semi-standard stationary phases [19]. Data processing in this case is illustrated by Table 3, similarly to Table 2. The parameters of the equations (RI_ref_ − RI_exp_) = *a*RI_exp_ + *b* are presented in the footnotes.

The statistical characteristics of the data sets in Table 2 and Table 3 are close to each other, as they should be according to the recommendations mentioned above. For the second of them, the average value of the difference Δ_ref-exp_ for the complete initial data set is 8 i.u., the average value of Δ_corr-ref_ for the corrected values is 5 ± 4 i.u., and for the reduced data set it is 6 ± 4 i.u. However, this example illustrates the possibility of identifying compounds characterized by RIs on standard nonpolar phases using reference data for semi-standard nonpolar phases. The algorithms of data processing are the same in both cases.

Among the 32 compounds in Table 3, only 1, myrcene, does not meet the criterion (7). The values of RI_corr_ and RI_corr_ * for myrcene differ from reference RIs [19] by more than 2*S*_0_; the cause of this difference remains unclear.

**Figure 4:**
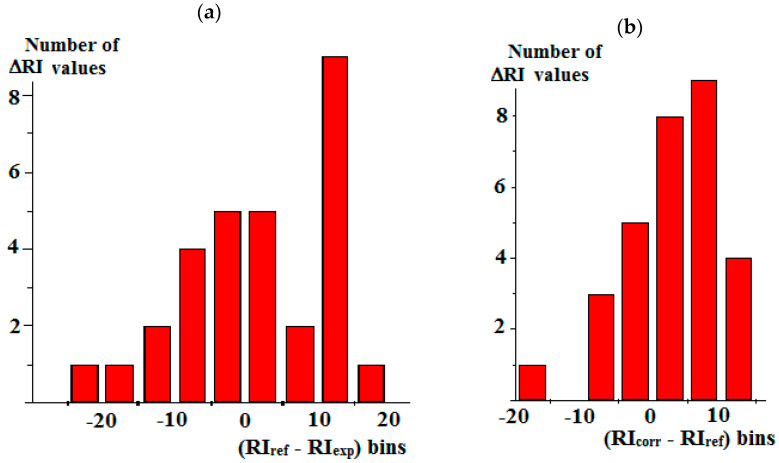
Histograms illustrating the results of comparing the RI data for essential oil components [22] with the reference data [19] for semi-standard nonpolar stationary phases (**a**) before and (**b**) after their correction. The bin sizes for both histograms are 5 i.u.

Similarly to Figure 3 (above), it seems reasonable to characterize the distributions of Δ_ref-exp_ and Δ_corr-ref_ for the reduced data set to the corresponding histograms. Figure 4a illustrates the spread of the initial differences (RI_ref_ − RI_exp_); it is rather asymmetric, and most of the values are located within the range from −15 to +15 i.u. The distribution of the (RI_corr_ − RI_ref_) values (b) corresponds to the data of Table 3 for comparing the RI data measured on standard nonpolar phases with the reference data for semi-standard phases and, moreover, calculated for the reduced data set. It is only slightly narrower (from −10 to +15 i.u.), but it becomes much more symmetric.

Hence, the algorithm proposed minimizes the shift in the differences in the initial experimental and reference RI values relative to zero and makes the distribution of these values narrower and more symmetric.

An important illustration of this algorithm is the situation when the reference RI values are determined under conditions close to those of the experimental determination of the RI values. It arises when the types of stationary phases are the same and the parameters of the GC column and temperature conditions are close each other. In these cases, both coefficients “*a*” and “*b*” of Equation (3) are obviously close to zero. To confirm this, let us consider the last table, Table 4, which illustrates the results of the experimental testing of the algorithm using as an example a simple (only 10 constituents whose content exceeds 0.1% of the total peak area) sample of *Lavandula angustifolia* essential oil. Both experimental and reference data relate to the same semi-standard nonpolar stationary phases: HP-5 (experimental) and DB-5 (reference [19]). Therefore, the coefficients of the equation (RI_ref_ − RI_exp_) = *a*RI_exp_ + *b* (3) appear to be minimal among all examples considered above *a* = 0.008 and *b* = −8.5; the difference Δ_corr-ref_ = RI_corr_ − RI_ref_ is only 2 ± 1, and the sum of residuals is *S*_0_ = 5.

The list of compounds in Table 4 includes two minor sesquiterpenes, C_15_H_24_, with retention indices of 1365 and 1384, which remain unidentified. The application of the algorithm considered provides corrected values of their retention indices, namely, 1361 and 1380. Hence, in the reference set of data [19], we should find sesquiterpenes with RIs within the intervals 1356–1366 and 1375–1385. Screening in the first of them leads to the rather “exotic” silfiperfol-4,7(14)-diene with an RI of 1358. However, an analog of this compound previously was identified in some species of *Lavandula* genus [23], and the RI of silfiperfol-4,7(14)-diene determined in [24] (1367) is also close to RI_corr_ in Table 5. However, the identification of this compound should be considered as tentative.

For the next sesquiterpene with an RI within the interval 1375–1385, according to databases [2,19], there are several possible candidates:

Let us additionally take into account the number of averaged RI values available for every compound in database [2] for standard/semi-standard nonpolar phases. It allows us to use such auxiliary (probability) criterion as the number of previous mentions of a particular compound [25]. Four compounds with the suitable RI from [19] appear to be little mentioned compared to α-copaene with an RI of 1376 [2] (377/698). However, this identification should also be considered as tentative.

## 3. Materials and Methods

The principal feature of this work is the possibility of using a practically unlimited number of examples taken from the literature. However, for special consideration we selected the RI values of alkylarenes on Apiezon L phase [20] (can be classified as semi-standard), data for essential oil constituents on standard DB-1 nonpolar phase [22], and experimental data for the essential oil of *Lavandula angustifolia* L. on semi-standard HP-5 phase. The reference RI data for the standard and semi-standard nonpolar phases were taken from the NIST17 database [2].

*The sample of Lavandula angustifolia essential oil* (Technical Specification 20.53.10-006-74840603-2018, Mirrolla Lab., Leningrad district, Kuzmolovskii Village, Russia, *n*_D_^20^ 1.4568) was purchased in a regular pharmacy. Isobutyl alcohol was of chemically pure grade (GOST (State Standard No. 6016-77, “Isobutyl alcohol”, Angarsk Chemical Reagent Plant, Angarsk, USSR); the reference *n*-alkanes C_7_–C_18_ were of chemically pure grade for chromatography (Reakhim, Moscow, Russia).

*Gas chromatographic analysis* of *Lavandula angustifolia* essential oil was carried out using its 10% solution in isobutyl alcohol with a Khromatek-Kristall 5000.2 gas chromatograph (Yoshkar-Ola, Russia) equipped with a flame ionization detector and a WCOT capillary column (length 10 m, internal diameter 0.53 mm, semi-standard HP-5 stationary phase, and film thickness 2.65 μm). Such column appears to be suboptimal for accurate analysis of essential oils (narrow-bore columns are preferable), but the reason of its selection was just the necessity of testing the algorithm. The analysis was performed with programmed heating from 90 to 240 °C, ramp 6 deg min^−1^. The carrier gas was nitrogen, with a flow rate of 3.8 mL min^−1^, linear velocity of 34 cm s^−1^, and the split ratio was 1:3. The injector and detector temperature was 200 °C. A 10 μL microsyringe volume was used for injecting the 1.0 μL samples. The solution of *n*-alkanes C_7_–C_18_ for determining the retention indices was injected separately.

Before *GC–MS analysis*, the previous sample of *Lavandula angustifolia* essential oil was diluted 100-fold with the same solvent. The analysis was performed with a Shimadzu QO 2010 SE gas chromatograph–mass spectrometer (Kyoto, Japan) equipped with an Optima 5 MS GC column (Macherey-Nagel GmbH, Duren, Germany), with a of length 30 m, internal diameter of 0.32 mm, and film thickness of 0.25 μm, with programmed heating in the range 90–270 °C, ramp 6 deg min^−1^. The carrier gas was helium and the flow rate was 1.82 mL min^−1^, linear velocity was 53.3 cm s^−1^, and the split ratio was 1:10. The injector temperature was 200 °C, and the interface and ion source temperature was 250 °C. The ionization energy was 70 eV, the mass range was 40–500 Da, and the chromatogram recording start delay time was 2.0 min.

*Processing and presentation of the results.* Excel (Microsoft Office 2010) and Origin (versions 4.1 and 8.1) software were used for the statistical data processing and construction of the histograms. The QBasic (Version 1.1) program was used for calculating the linear-logarithmic RIs [1].

## 4. Conclusions

The suggested algorithm for comparing the experimental and reference GC retention indices as an important element of chromatographic and GC–MS identification of organic compounds is aimed at eliminating a significant element of uncertainty inherent in many contemporary recommendations: the use of fixed limiting differences between the experimental and reference GC retention indices, ΔRI = (RI_ref_ − RI_exp_) ≤ RI_lim_. The algorithm is effective for complex multicomponent mixtures in which at least several reliably identified components with known reference RI values can be revealed. It includes calculating the coefficients of regression equations for adaptive limits ΔRI = (RI_ref_ − RI_exp_) = *a*RI_exp_ + *b*, followed by using these relations for recalculating of all the other RI values into corrected data RI_corr_ = RI_exp_ + ΔRI. This algorithm allows for the interpretation of retention indices measured on standard nonpolar polydimethylsiloxane stationary phases using reference data for semi-standard nonpolar phases like polydimethylsiloxanes containing 5% phenyl groups and vice versa. It is applicable both to the statistical processing of reference data (presented in the format “average arithmetic value ± permissible deviation”) and to the results of single determinations. Furthermore, it allows us to use different RI databases and, if necessary, compare different databases with each other and even recalculate the reference data from one database (e.g., semi-standard) into another one (standard).

The necessity of the corrections of GC retention times and/or indices before operating with them and the factors influencing their values sometimes was discussed (see, e.g., [26]). However, the results of the current work indicated that such corrections might be recommended not as exceptions, but as a regular step of data processing during RI library searches.

## Figures and Tables

**Table 1:** Comparison of the retention indices published in [20] with RI values from NIST database [2] on standard nonpolar polydimethylsiloxane stationary phases.

Compound	RI_exp_ [20]	RI_ref_ [2]	Δ_ref-exp_	Complete Set of Reference Data		Reduced Set of Reference Data	
				RI_corr_	Δ_corr-ref_	RI_corr_ *	Δ_corr-ref_
**Benzene**	**679**	**654 ± 7**	**−25**	**650**	**−4**	**650**	**−4**
**Toluene**	**790**	**757 ± 6**	**−33**	**761**	**+4**	**760**	**+3**
Ethylbenzene	879	850 ± 6	−29	850	0	849	−1
*m*-Xylene	893	860 ± 6	−35	866	+6	865	+5
*p*-Xylene	893	860 ± 6	−33	864	+4	863	+3
*o*-Xylene	919	881 ± 6	−37	890	+9	889	+8
Isopropylbenzene	934	919 ± 7	−15	905	−14	904	−15
Propylbenzene	966	945 ± 5	−21	937	−8	936	−9
1-Methyl-4-ethylbenzene	983	953 ± 5	−30	954	+1	953	0
*tert*-Butylbenzene	998	986 ± 7	−12	969	−17	968	−18
1-Methyl-2-ethylbenzene	999	969 ± 5	−30	970	+1	969	0
**1,3,5-Trimethylbenzene**	**1002**	**962 ± 6**	**−40**	**974**	**+12**	**972**	**+10**
*sec*-Butylbenzene	1019	1000 ± 5	−19	991	−9	989	−11
1,2,4-Trimethylbenzene	1027	983 ± 5	−44	999	+16	997	+14
1,3-Diethylbenzene	1054	1040 ± 5	−14	1026	−14	1023	−17
1,2,3-Trimethylbenzene	1058	1010 ± 6	−48	1030	+20	1027	+17
**Butylbenzene**	**1068**	**1047 ± 6**	**−21**	**1040**	**−7**	**1037**	**−10**
1-Methyl-2-propylbenzene	1076	1058 ± 5	−18	1048	−10	1045	−13
**1,2,4,5-Tetramethylbenzene**	**1139**	**1107 ± 5**	**−32**	**1111**	**+4**	**1108**	**+1**
1,2,3,5-Tetramethylbenzene	1152	1110 ± 6	−42	1124	+14	1121	+11
Average standard deviation of reference RI values, *s*_RI_		5.8					
Average difference Δ_ref-exp_:			−29 ± 10				
Average difference Δ_corr-ref_:					9 ± 6 (0 ± 11) **		8 ± 6 * (−1 ± 10) **

Footnotes: The names and numerical data for compounds included in the reduced data set are marked in bold; (*) RI_corr_ and Δ_corr-exp_ values are calculated using the reduced data set; (**) average values ± standard deviations considered by taking into account the signs of the initial data are indicated in parentheses. The parameters of the equation Δ_ref-exp_ = RI_ref_ − RI_exp_ = *a*RI_exp_ + *b* for the complete data set are *a* = 0.002 ± 0.024, *b* = −30.5 ± 23.3, *R* = −0.02, and *S*_0_ = 10.8, and for the reduced data set, *a* = −0.005 ± 0.021, *b* = −25.3 ± 20.6, *R* = −0.14, and *S*_0_ = 8.4.

**Table 2:** Comparison of the retention indices of essential oils constituents [22] with RI values from the NIST database [2] on standard nonpolar polydimethylsiloxane stationary phases.

Compound	RI_exp_	RI_ref_ [2]	Δ_ref-exp_	Complete Set of Reference Data		Reduced Set of Reference Data	
				RI_corr_	Δ_corr-ref_	RI_corr_ *	Δ_corr-ref_ *
α-Pinene **	**950**	**933 ± 4**	**−17**	**936**	**+3**	**930**	**−3**
Camphene	969	946 ± 5	−23	956	+10	950	+4
Myrcene	985	983 ± 3	−2	970	−15	967	−18
3-Carene	1022	1006 ± 5	−16	1010	+4	1005	−1
**Limonene**	**1038**	**1017 ± 3**	**−21**	**1026**	**+9**	**1022**	**+5**
**Linalool**	**1089**	**1086 ± 3**	**−3**	**1077**	**−9**	**1076**	**−10**
Isofenchol	1114	-	-	-	-	-	-
Fenchone	1120	1105 ± 6	−15	1109	+4	1108	+3
Citronellal	1138	1134 ± 4	−4	1127	−7	1127	−7
(*Z*)-Verbenol	1144	1133 ± 5	−11	1133	0	1148	+15
**Camphor**	**1146**	**1123 ± 6**	**−23**	**1135**	**+12**	**1136**	+13 *
(*Z*)-Pinocarveol ***	1146	1135 ± 7	−11	1135	0	1136	+1
		1126 ± 6	−20	1135	+9	1136	+10
(*E*)-Verbenol	1147	1133 ± 5	−14	1136	+3	1137	+4
Isopulegol	1148	1144 ± 5	−4	1137	−7	1138	−6
(*Z*)-Pinocamphone	1160	1140 ± 5	−20	1149	+9	1150	+10
Borneol	1171	1151 ± 18	−20	1161	+10	1162	+11
Menthol ***	1174	1157 ± 2	−17	1164	+7	1165	+8
		1165 ± 6	−9	1164	−1	1165	0
Terpinen-4-ol	1178	1164 ± 5	−14	1168	+4	1169	+5
Carenol	1188	-	-	-	-	-	-
α-Terpineol	1189	1175 ± 5	−14	1179	+4	1181	+6
Neocarveol	1189	-	-	-	-	-	-
Myrtenol	1195	1181 ± 5	−14	1185	+4	1187	+6
Verbenone	1202	1184 ± 7	−18	1092	+8	1194	+10
**Neral**	**1223**	**1218 ± 5**	**−5**	**1213**	**−5**	**1216**	**−2**
Carvone	1230	1218 ± 6	−12	1220	+2	1227	+9
Geraniol	1238	1237 ± 4	−1	1229	−8	1232	−5
Linalyl acetate	1242	1241 ± 2	−1	1233	−8	1236	−5
**Geranial**	**1250**	**1249 ± 8**	**−1**	**1241**	**−8**	**1245**	**−4**
Safrol	1275	1269 ± 7	−6	1266	−3	1271	+2
Bornyl acetate	1280	1270 ± 5	−10	1271	+1	1276	+6
(*Z*)-Pinocarvyl acetate	1301	-	-	-	-	-	-
Isosafrole	1357	1327 ± 31	−30	1349	+22 ^4^*	1357	+30 ^4^*
		1358 ± 6 ^5^*	+1	1349	**−9**	1357	**−1**
Average standard deviation of reference RI values, *s*_RI_		6.3					
Average difference Δ_ref-exp_:			13.2				
Average difference Δ_corr-ref_:					7 ± 5		6 ± 4

Footnotes: Dash means no information in database [2]; (*) RI_corr_ and Δ_corr-exp_ values are calculated using the reduced data set; (**) the names and numerical data for compounds included in the reduced data set are marked in bold; (***) compounds with two different RI values in database [2]; (^4^*) isosafrole is the single compound for which the values of ΔRI_corr_ for both complete and reduced data sets differ from the average values of RI_corr_ − RI_ref_ by more than two standard deviations (see comments in the text); (^5^*) RI value for β-isosafrole from author’s RI collection. The parameters of the equation Δ_ref-exp_ = RI_ref_ − RI_exp_ = *a*RI_exp_ + *b* for the complete data set are *a* = 0.014 ± 0.021, *b* = −26.8 ± 24.2, *R* = 0.13, and *S*_0_ = 11.6, and for the reduced data set are, *a* = 0.049 ± 0.035, *b* = −66.6 ± 39.6, *R* = 0.57, and *S*_0_ = 9.0.

**Table 3:** Comparison of the retention indices of essential oils constituents [22] with the reference RI values from [19] for semi-standard nonpolar stationary phases.

Compound	RI_exp_	RI_ref_ [19]	Δ_ref-exp_	Complete set of Reference Data		Reduced set of Reference Data	
				RI_corr_	Δ_corr-ref_	RI_corr_ *	Δ_corr-ref_ *
α-Pinene **	**950**	**932**	**−18**	**933**	**+1**	933	+1
Camphene	969	946	−23	953	+7	954	+8
Myrcene	985	988	+3	970	−18 ***	972	−16 ***
3-Carene	1022	1008	−14	1010	+2	1012	+4
**Limonene**	**1038**	**1024**	**−14**	**1028**	**+4**	1029	+5
**Linalool**	**1089**	**1095**	**+6**	**1083**	**−12**	1085	−10
Isofenchol	1114	1114	0	1110	−4	1112	−2
Fenchone	1120	1118	−2	1116	−2	1119	+1
Citronellal	1138	1148	+10	1136	−12	1139	−9
(*Z*)-Verbenol	1144	1137	−7	1142	+5	1145	+8
**Camphor**	**1146**	**1141**	**−5**	**1144**	**+3**	1147	+6
(*Z*)-Pinocarveol	1146	1135	−11	1144	+9	1147	+12
(*E*)-Verbenol	1147	1140	−7	1145	+5	1148	+8
Isopulegol	1148	1145	−3	1146	+1	1149	+4
(*Z*)-Pinocamphone	1160	1172	+12	1159	−13	1163	−9
Borneol	1171	1165	−6	1171	+6	1175	+10
Menthol	1174	1167	−7	1174	+7	1178	+11
Terpinen-4-ol	1178	1174	−4	1179	+5	1182	+8
Carenol	1188	-	-	-	-	-	-
α-Terpineol	1189	1189	0	1191	+2	1194	+5
Neocarveol	1189	-	-	-	-	-	-
Myrtenol	1195	1194	−1	1197	+3	1201	+6
Verbenone	1202	1204	+2	1205	+1	1208	+4
**Neral**	**1223**	**1235**	**+12**	**1227**	**−8**	1231	−4
Carvone	1230	1239	+9	1235	−4	1239	0
Geraniol	1238	1249	+11	1244	−5	1248	−1
Linalyl acetate	1242	1254	+12	1248	−6	1252	−2
**Geranial**	**1250**	**1264**	**+14**	**1257**	**−7**	1261	−3
Safrol	1275	1285	+10	1284	−1	1288	+3
Bornyl acetate	1280	1284	+4	1289	+5	1293	+11
(*Z*)-Pinocarvyl acetate	1301	1311	+10	1312	+1	1316	+5
**Isosafrole**	**1357**	**1373**	**+16**	**1372**	**−1**	1377	+4
Average difference Δ_ref-exp_:			8.4				
Average difference Δ_corr-ref_:					5 ± 4		6 ± 4

Footnotes: Dash means no information in [19]; (*) RI_corr_ and Δ_corr-exp_ values here are calculated using the reduced data set; (**) the names and numerical data for compounds included in the reduced data set are marked in bold; (***) myrcene is the single compound for which ΔRI_corr_ values for both complete and reduced data sets differ from the average values of RI_corr_ − RI_ref_ by more than two standard deviations. The parameters of the equation RI_ref_ − RI_exp_ = *a*RI_exp_ + *b* for the complete data set are *a* = 0.080 ± 0.014, *b* = −93.4 ± 16.5, *R* = 0.72, and *S*_0_ = 7.8, and for the reduced data set are, *a* = 0.091 ± 0.019, *b* = −103.0 ± 22.4, *R* = 0.90, and *S*_0_ = 6.5.

**Table 4:** Comparison of the retention indices of components of *Lavandula angustifolia* essential oil with reference data from [19] (all RI values were measured on semi-standard nonpolar stationary phases).

	RI_exp_	RI_ref_ [19]	ΔRI_ref-exp_	RI_corr_	ΔRI_corr_
Camphene	949	946	−3	948	+2
Myrcene	993	988	−5	992	−1
(*E*)-Ocimene	1038	1044	+6	1037	−1
γ-Terpinene	1049	1054	+5	1048	−1
Linalool	1100	1095	−5	1098	+3
Alloocimene	1136	1128	−8	1134	−2
Linalyl acetate	1256	1254	−2	1253	−3
C_15_H_24_ *	1365	-	-	1361	-
C_15_H_24_ *	1384	-	-	1380	-
Aromadendrene	1443	1439	−4	1439	−4
Average difference Δ_ref-exp_:			4.8		
Average difference Δ_corr-ref_:					2 ± 1

Footnotes: (*) For identification of the sesquiterpenes, see discussion in the text. Parameters of the equation ΔRI_ref-exp_ = *a*RI_ref_ + *b*: *a* = −0.007 ± 0.012, *b* = 6.0 ± 13.9, *R* = −0.23, and *S*_0_ = 5.2.

**Table 5:** Comments to the identification of two sesquiterpenes in Lavandula angustifolia essential oil (see Table 4).

RI	Compound	Number of RI Values for Standard/Semi-Standard Phases in [2]
1376 [2]	α-Copaene	377/698
1377 [19]	Silfiperfol-6-ene	None
1379 [19]	β-Patchoulene	10/23
1380 [19]	Daucene	16/14
1381 [19]	β-Panansinene	1/5

## Data Availability

The original contributions presented in this study are included in the article. Further inquiries can be directed to the corresponding author.
